# Congenital Amusia Persists in the Developing Brain after Daily Music Listening

**DOI:** 10.1371/journal.pone.0036860

**Published:** 2012-05-11

**Authors:** Geneviève Mignault Goulet, Patricia Moreau, Nicolas Robitaille, Isabelle Peretz

**Affiliations:** The International Laboratory for Brain Music and Sound Research (BRAMS), Department of Psychology, University of Montreal, Montreal, Québec, Canada; Northwestern University, United States of America

## Abstract

Congenital amusia is a neurodevelopmental disorder that affects about 3% of the adult population. Adults experiencing this musical disorder in the absence of macroscopically visible brain injury are described as cases of congenital amusia under the assumption that the musical deficits have been present from birth. Here, we show that this disorder can be expressed in the developing brain. We found that (10–13 year-old) children exhibit a marked deficit in the detection of fine-grained pitch differences in both musical and acoustical context in comparison to their normally developing peers comparable in age and general intelligence. This behavioral deficit could be traced down to their abnormal P300 brain responses to the detection of subtle pitch changes. The altered pattern of electrical activity does not seem to arise from an anomalous functioning of the auditory cortex, because all early components of the brain potentials, the N100, the MMN, and the P200 appear normal. Rather, the brain and behavioral measures point to disrupted information propagation from the auditory cortex to other cortical regions. Furthermore, the behavioral and neural manifestations of the disorder remained unchanged after 4 weeks of daily musical listening. These results show that congenital amusia can be detected in childhood despite regular musical exposure and normal intellectual functioning.

## Introduction

The wide use of digital media has made music pervasive, especially in childhood and adolescence. The potential effect of such extensive music exposure raises fundamental questions regarding the plasticity of auditory brain mechanisms. Indeed, daily music listening is capable of changing brain activity and cognitive recovery after a stroke [1], [2]. However, little is known about the consequences of such musical enrichment in a normal and developing brain. In the animal brain, the enriched environment can induce plastic changes ranging from biochemical parameters to dendritic arborization, gliogenesis, neurogenesis, and improved learning [3]. In the auditory domain, frequent exposure to complex sounds, such as music, improves the response strength, selectivity and latency of auditory cortex neurons [4] and leads to learning (e.g., [5]). Accordingly, in humans, regular musical stimulation can create an enriched environment, which has the potential to enhance brain plasticity at multiple levels, influencing both auditory functions and learning mechanisms [6]. Here, we examine whether such beneficial effects of music stimulation extend to children with congenital amusia.

Congenital amusia is a neurodevelopmental disorder that is characterized by a deficit in melody processing that cannot be explained by brain damage or intellectual deficiencies [7]. This musical disorder is hereditary [8], [9]. It is termed “congenital” under the assumption that normal musical development never took place and that the condition was therefore present from birth^1^.

The implication is that congenital amusia should be observed in childhood. The disorder has been found to be present in 7–9 year old children who also tested abnormal on a screening test for dyslexia [10]. However, the disorder should be observed in relative isolation from other cognitive deficits as documented in adults. We recently reported such a case in a 10-year-old child who exhibited poor melody discrimination and poor singing, despite early and regular choir lessons [11]. One possibility is that this amusic child was avoiding musical solicitation outside the imposed choir participation. Such a form of musical deprivation may in turn aggravate the musical handicap. In contrast, the constant musical exposure that is typical of the young generation may protect the majority of children from expressing congenital amusia. The goal of this study was to test for the presence and robustness of amusia in a group of children who listened to music daily.

We tested eight amusic children and eight typical peers comparable in age, socio-economic background and intellectual functioning on behavioral and brain measures that are well-documented signatures of the adult form of amusia. Congenital amusia is usually diagnosed with the Montreal Battery of Evaluation of Amusia (MBEA; [12]). The battery comprises six tests (180 items in total) assessing the different components of melody processing in Western tonal music, namely pitch contour, musical scales, pitch intervals, rhythm, meter and memory. Typically, an individual whose global score (averaged across the six tests) lies two standard deviations below the mean of normal controls is considered amusic. The test that is most diagnostic of amusia is the MBEA scale test, which requires participants to discriminate between pairs of melodies in which one contains a single out-of-key tone. (e.g., [13], [14]). This musical pitch disorder represents a clear-cut phenotype that has served to identify its neurogenetic origins [8].

The likely faulty mechanism of the musical pitch disorder lies in the acoustical encoding of pitch. Amusic individuals are impaired in detecting pitch direction [13], [15], [16] and pitch deviations smaller than two semitones in tone sequences [17] as well as in tone pairs [18]. Given that amusic individuals are probably born with such an elemental deficit (normal infants’ pitch acuity is in the order of half a semitone), they may have developed a poor understanding of musical structure. Support for this tight relation between fine-grained pitch discrimination and musical abilities can be found in the strong correlation between pitch acuity and the melodic tests of the MBEA, observed in a student population [19]. Thus, a perceptual system that is unable to detect small pitch changes is more likely to miss an essential part of musical structure [20].

At the neural level, we were able to identify an electrical brain marker for amusia. The (adult) amusic brain does not produce a normal P300 in response to the detection of small pitch deviations (25 and 50 cents; 1 semitone  = 100 cents) [21]. This altered pattern of electrical activity did not seem to arise from an anomalous functioning of the auditory cortex because the N100 component was normal. Rather, the ERPs might reflect difficulties that occur in later processing stages along the auditory pathway that involves frontal regions, since it seems to be related to the attentional demands of the task.

Attentional requirements (or awareness) seem to play a key role in the behavioral manifestation of congenital amusia. Using the same Event Related Potentials (ERPs) method, we showed that the adult amusic brain can track quarter-tone (50 cents) pitch differences in melodies and eighth-tone (25 cents) pitch differences in repeating tone sequences, as evidenced by an early right-lateralized negative brain response. This early negativity, or mismatch negativity (MMN), was obtained while participants either ignored the sounds [22] or failed to detect the pitch change at a behavioural (conscious) level [23]. The findings have been replicated in a recent functional magnetic resonance imaging (fMRI) study in which amusics were scanned while passively listening to pure-tone melodic patterns in which the successive tones differed in small steps from zero to two semitones [24]. Both amusic and control participants showed a positive linear BOLD response as a function of increasing pitch distance (including 25 and 50 cents) in bilateral auditory cortices (on the border of the planum temporale and lateral Heschl giri).

The relatively normal functioning of the auditory cortical responses to pitch changes in amusia supports the initial suggestion that the principal functional abnormality may lie outside the auditory cortex. In the fMRI study [24], we observed an abnormal response in the pars orbitalis of the right inferior frontal gyrus (IFG; BA 47/11). The amusic participants showed a decreased BOLD activation in the right IFG compared to control participants, who had a slightly increased BOLD activation relative to baseline in the same region. Furthermore, the auditory cortex was functionally connected to the right IFG in the normal brain but showed decreased functional connectivity in the amusic brain. These findings indicate an abnormal propagation of sequential pitch information between the auditory cortex and the right inferior frontal cortex in the adult amusic brain.

Here, we tested amusic children for the same behavioral and electrical brain markers found in amusic adults. We expected to find the same pattern of electrophysiological effects in the amusic children, with the possible exception of one component –the MMN–because it was absent in the previously published single case study of amusia in childhood [11]. This component is of particular interest in the developing brain for multiple reasons. The amplitude of the MMN can index impaired auditory discrimination of speech sounds in children [25]. Moreover, the MMN can be a precursor of neural plasticity, at least in adults. Tremblay and collaborators [26] observed that speech-sound discrimination training in adults increased the MMN, which always preceded or accompanied improvements seen in behavior. Finally, regular music listening sessions have been shown to enhance the amplitude of the MMN in response to pitch deviations in normal children [27].

We measured the P200 responses to tones as an electrophysiological marker of auditory training [28]. Increases in the amplitude of the P200 are brought about by auditory training and musical practice; they are considered to reflect an increased efficiency of the neural networks subserving pitch processing, either because more neurons are active or because their activity is more synchronized (e.g., [29]). These brain measures were expected to reveal the malleability of the auditory cortex in the amusic children.

## Results

**Figure 1:**
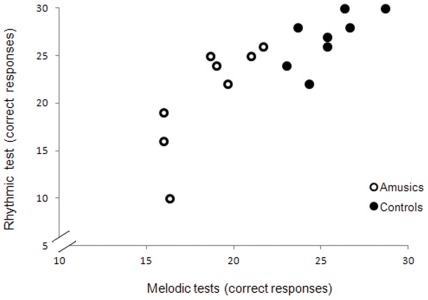
Melodic and rhythmic scores. Individual melodic scores (averaged across the scale, contour and interval tests) and rhythmic scores obtained on the MBEA. Maximum score is 30; chance = 15.

The eight amusic children obtained global scores between 54 and 76% correct on the MBEA [12], which were below the scores of the non-amusic controls (78–96%; see S1). As can be seen in Figure 1, all amusic children were impaired on the melodic (scale, contour and interval) tests, and three of them were also impaired on the rhythm test. This pattern is typical of amusic adults in both Western and Eastern countries (e.g., [30], [31]).

All children had normal verbal reasoning and concept formation as assessed by the similarities subtest of the Weschler Intelligence Scale for Children (WISC-IV). They showed normal visual perception and organization, concentration, and visual recognition of essential details of objects, as indexed by the picture completion subtests of the WISC-IV. They also had normal fluid intelligence, as assessed by the matrix reasoning, which is also a reliable estimate of general intellectual ability [32]. Individual results on these standard tests are presented along with demographic information in Table 1 and S1.

**Table 1:** Characteristics of participants and standard neuropsychological tests results.

		Demographic characteristics				WISC-IV ** MBEA				
		Age	Gender	Musical Training (private lessons)	Listening habits (hr/week)	Similarities	Matrix	Picture completion	Scale	
Amusics										
	1	10.8	f	No	1	11	9	8	14	
	2	11.8	f	No	2	8	12	10	15	
	*3	11.1	m	No	0.5	12	10	12	17	
	4	12.1	m	Yes (6 months guitar)	3	13	10	15	18	
	5	12.2	f	No	8	11	13	12	18	
	6	11.5	f	No	2	12	10	12	18	
	7	11.2	f	No	2	10	9	10	19	
	8	11.5	m	Yes (6 months violin)	8	14	14	11	19	
	***M***	**11.5**				**11.4**	**10.9**	**11.3**	**17.3**	
Controls										
	1	10.9	f	No	4.5	12	10	11	23	
	2	11.0	f	No	2	14	12	11	23	
	3	13.2	f	No	8	12	10	13	24	
	4	12.3	f	No	8	9	13	13	25	
	5	11.8	m	No	2	11	12	10	25	
	6	12.3	f	Yes (4 years piano)	8	15	10	13	26	
	*7	10.9	m	No	2	13	10	12	27	
	8	11.2	f	Yes (3 years flute, piano)	3	10	10	12	28	
	***M***	**11.7**				**12.0**	**10.9**	**11.9**	**25.1**	

* = Participants who were not re-tested.

Individual age, education, gender and music listening habits are presented along with the scaled scores obtained on the similarities, matrix and picture completion tests of the WISC-IV, and on the scale test of the MBEA (maximum score  = 30; chance score  = 15).

One amusic participant dropped out of the study after test; thus, the data of her matched control were excluded from the re-test phase of the study. The remaining seven amusic and seven control peers were retested four weeks later, after daily music listening. These 14 participants received an MP3 digital player (Nextar 2 GB) containing 200 songs selected from popular music websites (e.g., “No one”, by Alicia Keys). They were instructed to listen to the music for a minimum of 30 minutes each day, and to record the listening duration in a diary. We did not add any task so as to keep the listening experience both ecological and pleasurable. After 4 weeks of this assignment, the 14 children came back to the laboratory and were tested with the same electroencephalogram (EEG) procedures as before.

During the 4 weeks period between test and re-test, amusics reported listening to the music for 45.6 min per day on average (*SD* = 19.2) while their control peers reported 30.5 min per day on average (*SD* = 5.7). The difference failed to reach significance (*t*_12_ = 2.00; *p* = .07, by a bilateral test). There was no indication that this daily musical activity had an impact on melody discrimination as assessed with the MBEA scale test. Amusics scored as poorly (with 19.3 correct responses out of 30 on average) at re-test as at initial testing (17.3 correct responses, t_6_ = 0.903, *p* = .40). Furthermore, at re-test, the MBEA scale scores negatively correlated with music listening duration (*r* = 0.77, *d.f.* = 12, *p* **<**0.01). Thus, the poorer performance is, the more the child listens to music.

**Figure 2:**
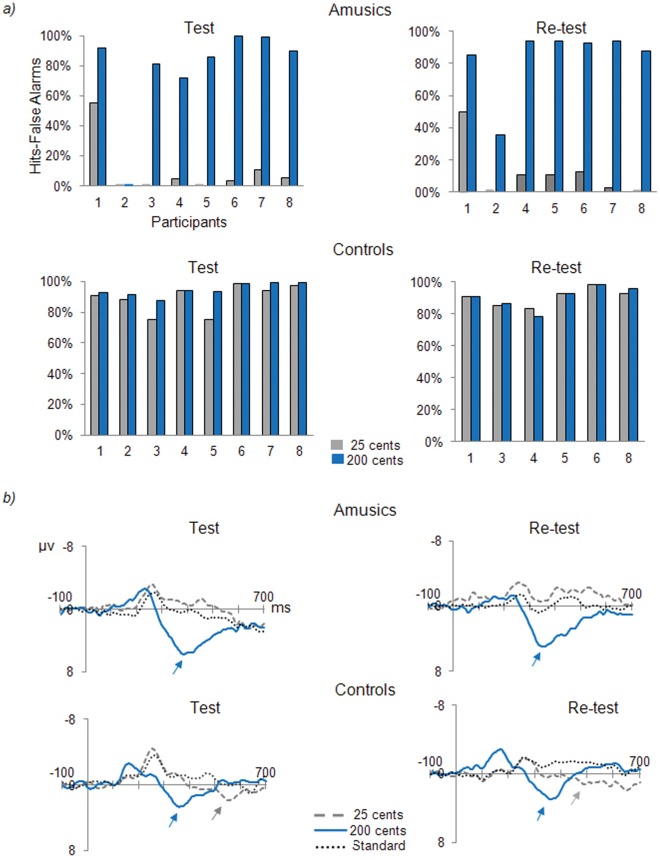
Pitch change detection and brain responses. *(*A) Individual percentage of hits-F.A.s in the detection of a changed pitch as a function of size. Behavioral data for control #2 at re-test are unavailable for technical reasons. (B) Grand-average ERPs for amusics and controls, time-locked to the onset of the fourth tone of the sequence, recorded at Pz in the pitch change detection task, before and after the daily music listening program. Arrows indicate the P300.

As predicted, the amusic difficulties in melody discrimination can be traced down to an acoustic pitch discrimination deficit (see Figure 2A at test). In the pitch change detection task, the amusic children detected the large (200 cents) pitch changes as accurately as their control peers (*t_14_ = *1.47, *p* = .16). This result shows that the two groups did not differ in terms of task comprehension or attention allocation. In contrast, the amusic children exhibited marked difficulties in perceiving the small (25 cents) pitch changes (*t_14_* = 10.80, *p*<.001, bilateral). This pattern was supported by a significant interaction between Group and pitch deviation Size, with *F*(1,14) = 27.40, *p*<.001. The results highlight the fine-grained nature of the pitch disorder, as observed in adults [17], [21].

This difference in behavior between amusics and controls is mirrored by the late positive component of the event-related potentials (ERPs; Figure 2 at pre-test). The (2×2) ANOVA including the factors of Group (amusics, controls) and pitch Size (25 cents, 200 cents), using the difference waves at Pz as a dependent variable revealed a significant interaction between Group and pitch Size, *F*(1,14) = 7.38, *p*<.05, which reflects the fact that the 25 cents pitch changes elicited a larger P300 in the control group as compared to the amusic group (*t_14_* = 1.82, *p*<.05, unilateral test). There was no difference in baseline since the two groups did not differ on the standard (repeated) tone (*t_14_ = *0.82, *p* = 0.42, bilateral test). The latency of the P300, its morphology, and its scalp distribution are consistent with the typical P300 brain potential observed in attentive, conscious detection of deviant pitches in acoustical contexts [33].

Fine-grained pitch discrimination was unchanged by 4 weeks of daily exposure to music. The amusic children did not improve their pitch detection performance (*t_6_* = 0.34 and 1.01, *n.s.*, for the 25 and 200 cents, respectively; Figure 2). The P300 remained absent for the 25 cents changes in the amusic brain and significantly smaller than the normal P300 (*t_12_* = 2.90, *p*<.01, unilateral test). Furthermore, the better was detection of the 25 cents changes, the larger was the P300, with *r* = 0.55, *d.f*. = 14, *p* = 0.03 at test, and *r* = 0.60, *d.f*. = 12, *p* = 0.03, at re-test.

**Figure 3:**
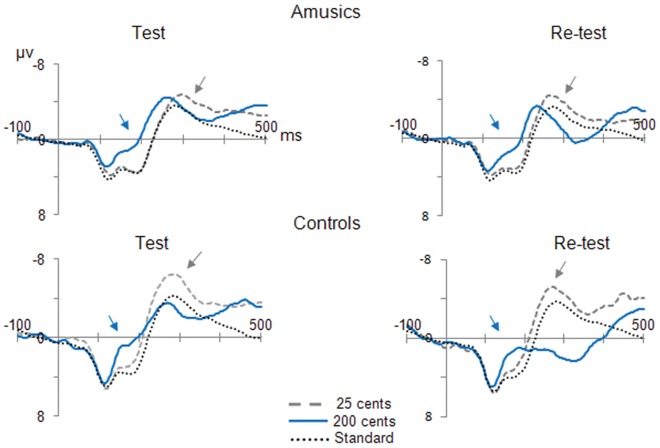
Preattentive brain responses to pitch changes. Grand-average ERPs for amusic and controls, in the MMN task, recorded at electrode Fz before and after the daily music listening program. Arrows indicate the MMN.

The anomalous brain response observed in amusics’ detection of fine-grained pitch differences did not extend to the MMN, as can be seen in Figure 3. As there was no difference between amusics and controls in their response to the standard tone (*t_14_* = 0.67, *p* = .52, bilateral test), the ANOVAs were conducted on the difference waves at Fz. The analyses revealed no significant difference between amusics and controls on the MMN (all *F<*1). In both groups, the MMN was delayed for the 25 cents pitch deviation as compared to the 200 cents pitch deviation (*F*(1,14) = 11.87, *p<*0.01). Note that the presence of a MMN for the 25 cents changes reached significance in amusics only (*t_7_* = 2.41, *p*<0.05, bilateral). The auditory cortex is the likely source of these MMNs, as there was an inversion of polarity at the mastoids [34].

At re-test, the MMN remained essentially unchanged (Figure 3). The presence of a MMN in response to the 25 cents deviation was replicated in amusics (*t*_6_ = 2.32, *p*<0.05, unilateral). The only difference to reach significance after 4 weeks of daily music listening was the MMN elicited by the large pitch (200 cents) deviation, which was reduced in control children (*t_6_* = 3.15, *p*<0.05). The decrease of the MMN was supported by an interaction between Time of testing (test vs. re-test) and pitch deviation Size (F(1,12) = 8.95, *p*<0.05). This might be due to an involuntary shift of attention for the salient pitch differences as indicated by the emergence of a late positivity in control children (Figure 3).

**Figure 4:**
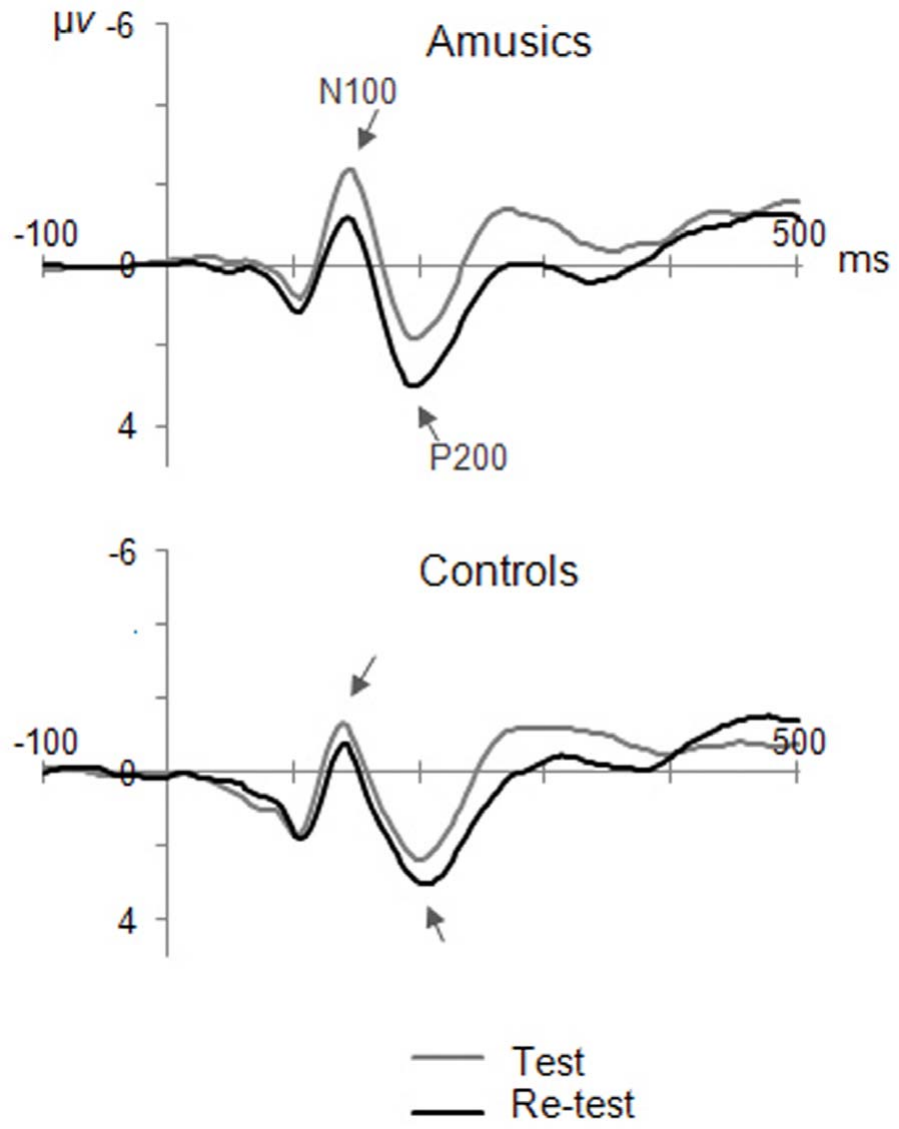
Preattentive brain responses to equally frequent tones. Grand-average ERPs for amusics and controls, elicited by the equally frequent tones, recorded at electrode Cz before and after the daily music listening program. Arrows indicate the N100 and the P200.

In order to assess the P200 component, three tones were presented equally often to the children while they watched the movie. As can be seen in Figure 4, the P200 at Cz was enhanced at re-test as compared to initial test, *F*(1,12) = 5.91, *p*<0.05. There was no Group effect nor interaction with Time of testing (*F*<1). Finally, the N100 preceding the P200 was not affected by Time of testing (*F*(1,12) = 4.34, *p* = 0.06), and did not differ between amusics and controls (*F* (1,12) = 1.43, *p* = 0.25).

## Discussion

The results show that the typical profile of congenital amusia can be observed in children despite daily music listening. The profile of amusia documented here in 10 to 13 year olds is strikingly similar to the profile studied at a more advanced age. The childhood expression of the disorder can be identified with the same screening tools –the MBEA- as those used with adults. Just like their older counterparts, amusic children exhibit difficulty in processing the melodic aspects of music, and rhythm to a lesser degree. Furthermore, the children were impaired at discriminating out-of-key notes within melodies. As in the adult form of amusia, the musical disorder is associated with a more elemental difficulty in detecting subtle pitch changes in repeating tones. This clear impairment in fine-grained pitch discrimination supports the notion that accurate pitch perception is foundational for the development of normal musical abilities [20].

The difficulty in small pitch change detection is mirrored in the brain responses. The amusic brain does not elicit a normal positivity (P300) in response to small pitch changes when they are undetected. This altered pattern of electrical activity does not seem to arise from an anomalous functioning of the auditory cortex, because all early components of the brain potentials, the N100, the MMN and the P200, appear normal. Rather, the brain and behavioral measures point to a later anomaly along the auditory pathway, probably in the connections with the frontal regions. These electrophysiological data reveal the same neural signature of amusia in the young brain as previously observed in the mature brain [21], [22], [24]. The dysfunction is highly consistent with the anatomical anomalies uncovered in the adult form of amusia. Adult amusics have abnormal grey and white matter in the inferior frontal cortex [30], [35], [36], and a marked decrease of fiber tracts in the right superior branch of the arcuate fasciculus [37]. Collectively, the evidence is consistent with a “functional disconnection” between a normally functioning auditory cortex and the inferior frontal gyrus.

Additional musical stimulation in the form of 30 minutes of daily music listening for one month does not affect the expression of congenital amusia. Thus, musical deprivation in childhood does not seem to be the cause of congenital amusia. The disorder is not only extremely stable and robust over one month, as shown here, but also over a lifetime since the profile of the disorder is identical to the one documented in the 50 to 70-year-old generation studied in our laboratory. This provides support for the qualification of the disorder as “congenital”.

Another major implication of the present findings is that additional musical stimulation by way of regular music listening is not an appropriate remediation strategy. We found no support for plastic changes related to daily music listening in the child brain. The only measure that changed after four weeks of regular music stimulation is the P200, which showed an enhancement in both the amusic and normal brain. The P200 component is thought to originate in the secondary auditory cortex [28] and its increase in amplitude is believed to reflect expanded cortical representation and enhanced tuning of neurons for the presented frequencies (after the animal work of [38]). It remains to be understood why such a change would affect the P200 selectively. Logically, the N100 and MMN should be similarly enhanced at re-test, since both ERP components are taken to reveal sensory encoding of sounds and both were measured in a passive listening procedure, like the P200. Future studies should aim at clarifying this issue by testing to what extent this P200 enhancement is specific to the presented (piano) tones or reflects frequency tuning.

Future remediation programs in congenital amusia should aim at training the pitch deficit. One attractive strategy would be to teach the child to sing or to play a musical instrument. Music making has long-term benefits not only for music processing but also for cognitive functioning [39]. Children who received one year of singing or keyboard lessons showed increased IQ, as compared to control groups who received drama lessons. Furthermore, learning to play a musical instrument shapes brain anatomy. Structural brain plasticity has been demonstrated in 6-year-old children following 15 months of weekly half-hour private keyboard lesson. Increases in size in motor and auditory areas (namely the right precentral gyrus, the corpus callosum and the right primary auditory region) were correlated with behavioral improvements on motor and auditory-musical tests [40].

Similarly, Moreno and collaborators [41] showed that 8-year-old children who received music lessons for six months showed improvements in reading and pitch discrimination while no such improvement was observed in children who received painting lessons. The behavioral enhancements in the musically trained children were associated with changes in the amplitude of the N300 elicited by fine-grained pitch discrimination (40 cents). This negativity enhancement may reflect the extra attention required to discriminate small changes in pitch [42]. Indeed, learning to play a musical instrument might train attention and concentration more than painting or music listening. However, similar plasticity effects have been observed in adult nonmusicians while attention requirements in the task were minimal. After two weeks of regular practice of musical arpeggios on a keyboard, an enhancement of the MMN was observed [43]. This enhancement was not observed in the control group who simply listened to and watched the keyboard exercises for two weeks. Thus, the attention required to practice a musical instrument may increase the number and activity of neurons that are tuned to the stimuli [28] and thereby may constitute an adequate remediation program for the amusic child. Enjoyment of such activities may also engage the child more fully, thereby increasing awareness and focused attention on musical structure.

Treating amusia and characterizing the neural changes associated with such interventions are important for both clinical and theoretical reasons. Clinically, it has the potential to improve the quality of life of the developing child. Indeed, music engagement may not only improve cognitive functioning but also social fitness, by improving social interactions [44] and cooperation [45]. Theoretically, characterizing the cognitive and neural changes associated with a successful intervention can provide further insight into the nature of the disorder, elucidate its plasticity, and inform models of normal auditory functioning.

Finally, it is important to note that the present findings have implications beyond congenital amusia. The musical disorder bears striking similarities to other neurodevelopmental disorders, such as congenital proposagnosia and developmental dyslexia. Like congenital amusia, the impairments affect a particular domain (face recognition or literacy) despite normal sensory perception, intellectual functioning and opportunities for acquiring the relevant skill. Like congenital amusia, these other disorders are hereditary [46], [47]. Like congenital amusia, a disconnection explanation has been offered for these disorders. Specifically, a disconnection account has been put forward for congenital proposagnosia, between the normally functioning ventral occipitotemporal cortex and other regions [48]. Prosopagnosic individuals have a reduction of white matter tracts that connect the ventral occipitotemporal cortex to anterior temporal and frontal cortices [49]. Similarly, developmental dyslexia has been attributed to reduced connectivity between temporal and parietal regions [50]. The similarities among these disorders suggest that a disruption caused by a genetic alteration, which disconnects the core systems of the circuit, can give rise to severe cognitive impairments. In sum, systematic research on congenital amusia may inform developmental disorders in general, not just musical development.

## Materials and Methods

### Participants

Eight amusic children and eight controls comparable in age (10 to 13 years), socio-economic background (Table S1) and musical training participated in the initial phase of the study. The amusic children were identified from a group of 300 children who took part in a large-scale evaluation of musical abilities in a school setting. In each group, two children had received formal extra-curricular musical training; however, the two amusics abandoned after 6 months. The two groups did not differ significantly in how many hours they reported listening to music (with 3.3 hrs/week on average in the amusic group and 4.7 hrs/week in the control group; Mann-Whitney *U* = 21, *z* = 1.21, *p* = 0.23). All children spoke French as their first language (except one control whose first language was English), were right-handers, with normal audiometry (below or at 20 dB HL from 500 Hz to 4000 Hz) and no known neuropsychological anomalies. Informed consent was obtained from the parents prior to the study. The protocol was approved by the Ethics Committee of the University of Montreal.

### Material and Procedure

All children were individually tested on the adult version of the MBEA [12] at the time of study. The whole battery, instructions, and stimulus examples can be found at www.brams.umontreal.ca/short/MBEA. The MBEA includes three melodic pitch-based tests (scale, contour, and interval), two time-based tests (rhythm and meter), and one memory test. The entire battery takes about an hour and a half to complete. The scale test was presented to the children before and after daily listening to music. It consists of 30 trials, with half containing pairs of identical melodies and half containing different pairs. When different, the comparison melody contains one pitch change that violates the key of the melody.

Children were seated in an electrically shielded and sound-attenuated chamber and EEG was continuously sampled with a Neuroscan system (Neuroscan SynAmps2; Compumedics, El Paso, TX) from 66 electrodes (bandpass, 0.5–70 Hz; sampling rate, 250 Hz), with cap reference. Bipolar electrode pairs monitored horizontal and vertical electrooculograms. All auditory stimuli were presented binaurally through Beyerdynamic headphones, with tones synthesized with a piano timbre at 70 dB SPL. The tones were 100 milliseconds (ms) long (with a rise and fall of 10 ms) and a 400 ms silent interval between tones (offset to onset) in each task, except for the P200 task for which there was a 2000 ms silent interval. The standard tone was played at a pitch level of C6 (1047 Hz). The pitch deviant tones were lower or higher in pitch than the standard tone by either 25 cents (1032 or 1062 Hz) or 200 cents (933 or 1175 Hz).

In the first task, tones were grouped in sequences of 5 in the pitch change detection task that was first performed, when attention was at its best. Each child was presented with 240 trials, each containing a sequence of 5 repeated tones. In half the trials, the fourth tone was changed upwards or downwards by 25 or 200 cents. Trials were randomized. The task was to press a “different” button whenever s/he detected a change and a “same” button when s/he was unable to hear a change. The task lasted about 15 minutes, without feedback. Before the task, the child was informed about the nature and position of the change, received 10 practice trials and was allowed to practice more than once if s/he wished to.

After the pitch change detection task, the child watched a self-selected silent movie with subtitles for about 45 minutes. During the first 20 minutes, three tones (the standard 1047 Hz, and the 1062 and 1175 Hz tones) were presented 150 times each, in a random order. The goal was to measure the N100 and P200. The presentation of these 450 tones was followed by the standard MMN procedure. The latter comprised 2736 standard tones (1047 Hz) and 76 tones added for each deviation type (creating a 10% probability of any deviance) in a random order with 400 ms silence between the tones. The children were instructed to watch the movie and to ignore the auditory stimulation, while their EEG activity continued to be recorded.

Offline, the EEG data were filtered (0.05–30 Hz, 24 dB/oct), re-referenced to the averaged potentials of the two mastoids and divided in epochs of 700 ms in the change detection task, and of 500 ms in the unattended condition. The 100 ms pre-stimulus interval served as the baseline, and artifacts were rejected (criterion: ±100 µV). The amplitude of the ERPs obtained for each target tone, regardless of performance, was quantified by computing the area under the curve in selected time windows. For the P300, the area under the curve was computed from 300–580 ms after tone onset, for the MMN from 140–400 ms, for the P200 from 160–260 ms and for the N100 from 80–160 ms. The latencies were taken at the time point of maximal potential in these time windows. The event-related potentials (ERPs) are reported at the electrode where they are the largest in both groups. The ERPs for upward and downward pitch changes were averaged together because pitch direction had no effect on either accuracy or brain responses.

## Supporting Information

Table S1 **Parents profession and MBEA average score.**
(DOCX)
