# Supplementary material for: Congenital Amusia Persists in the Developing Brain after Daily Music Listening
Source: PLoS One. 2012 May 11;7(5):e36860. doi: 10.1371/journal.pone.0036860 (PMC3350472; doi:10.1371/journal.pone.0036860)
Supplement: Table S1 — Parents profession and MBEA average score. (DOCX) [file pone.0036860.s001.docx]

|  | | Parent’s professions | MBEA average Score |
| --- | --- | --- | --- |
| Amusics | |  |  |
|  | 1 | Administrative assistant, Information technology manager | 20.3 |
|  | 2 | Delivery driver, Store manager | 16.5 |
|  | 3 | Police officer, Construction worker | 16.2 |
|  | 4 | Medical doctor, Computer systems analyst | 16.8 |
|  | 5 | Ventilation technician, Child care worker | 20.3 |
|  | 6 | Dietitian, Computer systems analyst | 20.5 |
|  | 7 | Team manager, Computer systems consultant | 22.3 |
|  | 8 | Engineer, Lawyer | 22.8 |
| Controls | |  |  |
|  | 1 | Cashier, N/A | 23,8 |
|  | 2 | Sales person , Translator | 23,3 |
|  | 3 | Teacher, Career counselor | 27,5 |
|  | 4 | Laboratory technician, CEGEP teacher | 23,8 |
|  | 5 | Executive assistant, Project manager | 23,7 |
|  | 6 | Treasurer, Volunteer | 28,2 |
|  | 7 | Chemist, Buyer for a company | 25,2 |
|  | 8 | Bioinformatics technician, Occupational rehabilitation | 28,7 |

**Supplementary Table. Parents profession and MBEA average score**

MBEA maximum average score = 30
